# Readiness To Manage Ebola Virus Disease Among Emergency Healthcare Workers in Uganda: A Nationwide Multicenter Survey

**DOI:** 10.21203/rs.3.rs-4212996/v1

**Published:** 2024-04-09

**Authors:** Ronald Olum, Bonaventure Ahaisibwe, Irene Atuhairwe, Thomas Balizzakiwa, Prisca Kizito, Mirriam Apiyo, Joseph Kalanzi, Assumpta Nabawanuka, Rony Bahatungire, Vanessa Kerry

**Affiliations:** Makerere University School of Public Health; Seed Global Health; Seed Global Health; Seed Global Health; Mbarara University; Case Hospital; Mulago National Referral Hospital; St Francis Hospital Nsambya; Ministry of Health; Harvard Medical School

**Keywords:** Ebola Virus Disease, Uganda, Readiness, Knowledge, Attitudes, Practices, Training Needs, Healthcare Workers

## Abstract

**Background:**

Emerging infectious diseases like the Ebola Virus Disease (EVD) pose significant global public health threats. Uganda has experienced multiple EVD outbreaks, the latest occurring in 2022. Frontline healthcare workers (HCWs) are at increased risk, yet there isn’t sufficient evidence of existing knowledge of EVD of these health workers. We aimed to assess the readiness of Uganda’s emergency healthcare workers to manage Ebola virus disease (EVD) and identify their training needs to inform targeted capacity-building interventions for future outbreaks.

**Methods:**

This multicentre nationwide cross-sectional study was conducted from July to August 2023 among 691 HCWs providing emergency care in 14 secondary and tertiary hospitals across Uganda. Participants were consecutively recruited using the probability-proportional-to-size sampling technique, and data was collected using a self-reported questionnaire. Factors associated with EVD knowledge were identified through a mixed-effect linear model.

**Results:**

Data from 691 eligible HCWs with a median age of 32 (IQR: 28–38) was analyzed (response rate: 92%). Only one-third (34.4%, n = 238) had received EVD training in the past year. The median EVD knowledge score was 77.4% (IQR: 71.2% − 83.4%). EVD knowledge was associated with longer professional experience in years (β: 0.21, 95% CI: 0.03 to 0.39, p = 0.024) and higher level of education: diploma (β: 3.37, 95% CI: 1.49 to 5.25, p < 0.001), undergraduate degree (β: 6.45, 95% CI: 4.11 to 8.79) and postgraduate degree (β: 7.13, 95% CI: 4.01 to 10.25, p < 0.001). Being a doctor (β: 2.55, 95% CI: 0.35 to 4.74, p = 0.023), providing care in the obstetrics/gynecology department (β: −1.90, 95% CI: −3.47 to − 0.32, p = 0.018), previous EVD training (β: 2.27, 95% CI: 0.96 to 3.59, p = 0.001) and accessing EVD information through social media (β: 2.52, 95% CI: 1.17 to 3.88, p < 0.001) were also significantly associated with EVD knowledge.

**Conclusion:**

Our study reveals that Ugandan HCWs’ EVD response readiness varies by individual factors and information sources. We recommend targeted training and suggest future research on educational innovations and social media’s potential to fill knowledge gaps.

## Introduction

Emerging and re-emerging infectious diseases pose a significant global public health challenge [1]. The viral outbreaks have markedly increased in the past two decades, including SARS-COV-1, Swine Flu, MERS-CoV, Ebola Virus Disease (EVD), and Zika [2]. These outbreaks extend beyond health implications, affecting social structures, economies, security, education, mobility, and food security [3]. Low-middle-income countries, burdened by existing health disparities, often bear the burden of these impacts. Uganda is one of the countries in Africa that has had the most viral hemorrhagic fever outbreaks, particularly EVD [4]. Since 2000, Uganda has experienced over five EVD outbreaks, the most recent between September 2022 and January 2023 [4–6]. The index case was a 24-year-old man from Mubende district in Western Uganda, who reportedly developed signs and symptoms of viral hemorrhagic fever and passed on nine days later [7]. This outbreak culminated in over 142 cases and 55 deaths, spreading across several districts, including Kyegegwa, Kassanda, Kagadi, Wakiso, Kampala, Masaka, Jinja, and Bunyangabu located in south-west, east and central regions of Uganda [8].

Lessons learned from previous disease outbreaks, notably the COVID-19 pandemic, have highlighted the critical need to strengthen healthcare systems worldwide [9, 10]. This includes enhancing human resources for health, financing, service delivery, health information systems, and access to medicines and leadership to respond to outbreaks effectively [9]. The COVID-19 crisis emphasized several challenges, notably a shortage of skilled health workers and a lack of essential medical supplies [11]. Unfortunately, this pandemic also saw a significant death of health workers globally [12]. The deadly 2014–2015 EVD outbreak in West Africa and previous EVD outbreaks in Uganda have had similar consequences [4, 5, 13]. By the end of the 2022 EVD outbreak in Uganda, 19 health workers had been confirmed to have contracted EBV, and 7 of them succumbed to the same [8]. Therefore, the readiness of the health workforce cannot be over-emphasized. Health workers are crucial in surveillance, case detection, and management, educating the population, and averting misinformation. Without adequate knowledge and skills in case detection, diagnosis, management, and infection prevention and control among emergency health workers, it is very challenging to control an outbreak.

Despite previous training on EVD in Uganda [14], recent studies, including one in Western Uganda, have identified significant knowledge gaps among healthcare workers, particularly in areas such as EVD biology, diagnosis, management, and prevention [15]. Similar challenges have been reported in some parts of Ethiopia [16], Pakistan [17], and Guinea [18]. There are also concerns about the readiness of healthcare facilities to manage EVD in Uganda, which in turn impacts how healthcare workers respond to the outbreak. In a study of 22 health facilities in the Kasese and Rubirizi districts, over half lacked essential resources such as budgets, case definition books, rapid response and burial teams for EVD management [15]. Furthermore, none of these facilities had dedicated EVD management centers, isolation units, or burial guidelines. Therefore, it is crucial that health workers are equipped with the necessary skills for diagnosing and managing patients with EVD, emphasizing the critical aspect of infection prevention and control. To effectively train health workers on EVD, it is important to assess what their current knowledge, and what their training needs are. The lack of recent studies in Uganda documenting these aspects poses a significant challenge in developing and implementing evidence-based training programs tailored to prepare healthcare workers for future outbreaks..

In this study, we aimed to evaluate the readiness of emergency healthcare workers in Uganda to manage Ebola virus disease. We also aimed to assess their training needs regarding EVD to inform targeted interventions to build capacity for future outbreaks.

## Methods

### Study design

This cross-sectional mixed-methods study, employing quantitative techniques, was conducted between July and September 2023.

### Study area

The study was conducted in 14 secondary and tertiary healthcare facilities across all the regions in Uganda. These included:

- Government-Owned Hospitals:
  - Mulago National Referral Hospital, located in Kampala, Central Region.
  - Jinja Regional Referral Hospital, situated in Jinja, Eastern Region.
  - Mbale Regional Referral Hospital, located in Mbale, Eastern Region.
  - Soroti Regional Referral Hospital, situated in Soroti, Eastern Region.
  - Moroto Regional Referral Hospital, located in Moroto, Northern Region.
  - Gulu Regional Referral Hospital, situated in Gulu, Northern Region.
  - Arua Regional Referral Hospital, found in Arua, Northern Region.
  - Hoima Regional Referral Hospital, located in Hoima, Western Region.
  - Fort Portal Regional Referral Hospital, situated in Kabarole, Western Region.
  - Kabale Regional Referral Hospital, located in Kabale, Western Region.
  - Mbarara Regional Referral Hospital, situated in Mbarara, Western Region.
- Private-Not-for-Profit (PNFP) Hospitals:
  - St Francis Hospital Nsambya, located in Kampala, Central Region.
  - St Mary’s Hospital Lacor, situated in Gulu, Northern Region.
- Private-For-Profit (PFP) Hospital:
  - Case Hospital, located in Kampala, Central Region.

### Study population

Health workers providing clinical services at emergency departments or those providing emergency services at the fourteen selected hospitals constituted the study population. These are usually the first contact with all patients seen and admitted at the hospital and are at a higher risk of encountering patients with Ebola viral disease. In many Ugandan hospitals, emergency care is provided across various departments, including those without a designated emergency room, often integrating emergency services within outpatient or inpatient wards for efficiency.

### Selection Criteria

We included nurses, clinical officers, junior house officers, medical officers, residents, and specialists aged 18 years and above who provided emergency care services to patients in the selected hospitals and consented to participate. Auxiliary staff or support health workers, undergraduate health profession students, and those critically ill or incapacitated to participate in the study were excluded.

### Sample size and sampling criteria

The sample size was calculated using Epi Info StatCalc (Centers for Disease Control and Prevention (CDC), Atlanta, Georgia, USA) sample size calculator. Using an expected frequency of emergency health workers with adequate knowledge of EVD at 50% since no similar study has been conducted in our setting, an acceptable margin of error of 5% and design effect of 2.0, a minimum of 754 participants are adequate to answer the research question, at 95% confidence intervals. The sample size was distributed proportionately across the 15 health facilities following a desk review of the current staffing norms at each level of care. Participants from each facility were recruited consecutively using convenience sampling until the desired sample size was reached.

### Data Collection Tool

A self-reported semi-structured questionnaire was used to assess knowledge, readiness, and training needs regarding the EVD (**Appendix 1**). The questionnaire was developed from information on the Ebola virus disease available on the CDC website (https://www.cdc.gov/vhf/ebola/index.html) and modified to suit the study population and setting. Some questions were also adopted from a previous study in Ethiopia [16]. The questionnaire consisted of participants’ sociodemographic data and questions on the basic science, clinical presentation, diagnosis, management, and prevention of EVD. Current practices regarding infection prevention and control were also assessed to determine readiness. Finally, the participants were asked to select the most important topics regarding EVD they would need training on.

### Study variables

#### Dependent variables:

Knowledge, attitudes, and practices towards Ebola virus disease. Knowledge was assessed using a 25-item questionnaire, attitudes using five 5-item Likert scale, and practices using five questions. Finally, three questions were used to determine the training needs on EVD among the participants.

#### Independent variables:

Social and demographic data (age, sex, level of education, professional cadre, hospital, and department), and EVD-related data (source of information on EVD, contact with an EVD patient, ever contracted EVD, ever isolated for EVD, prior EVD training).

### Data collection procedure

All potential participants were assessed for eligibility using the inclusion and exclusion criteria. All eligible participants were recruited after signing a written informed consent form. Each participant received an explanation and a copy of the questionnaire digitally designed using Kobo Toolbox (https://www.kobotoolbox.org/). The participants were then encouraged to fill in responses to all the questions in the data collection tool to ensure completeness and accuracy.

### Data Management Analysis

Completed questionnaires were exported to Microsoft Excel 365 for cleaning and coding. All statistical analyses were performed using STATA 18.0 software (StataCorp LLC, College Station, Texas, USA). To assess the level of knowledge, composite scores were calculated from the responses on knowledge of EVD and summarized as median (interquartile range). Attitudes, practices, and training needs were presented as frequencies and percentages. Factors associated with EVD knowledge were determined using a hierarchical linear regression model. Variables included in the model were selected based on extensive literature and those with p-values less than 0.2 with plausible explanations. The healthcare facility was included as a random effect, while individual-level parameters (age, sex, professional cadre, education, nature of residence (urban vs rural), professional experience, previous EVD training, and source of information) and hospital-level variables (geographical region, hospital ownership and departmental affiliations) were included as fixed effects. The results of the regression analyses were presented as beta coefficients (β) with 95% confidence intervals. A p-value of less than 0.05 was considered statistically significant.

## Results

### Sociodemographic Characteristics

**Figure 1:**
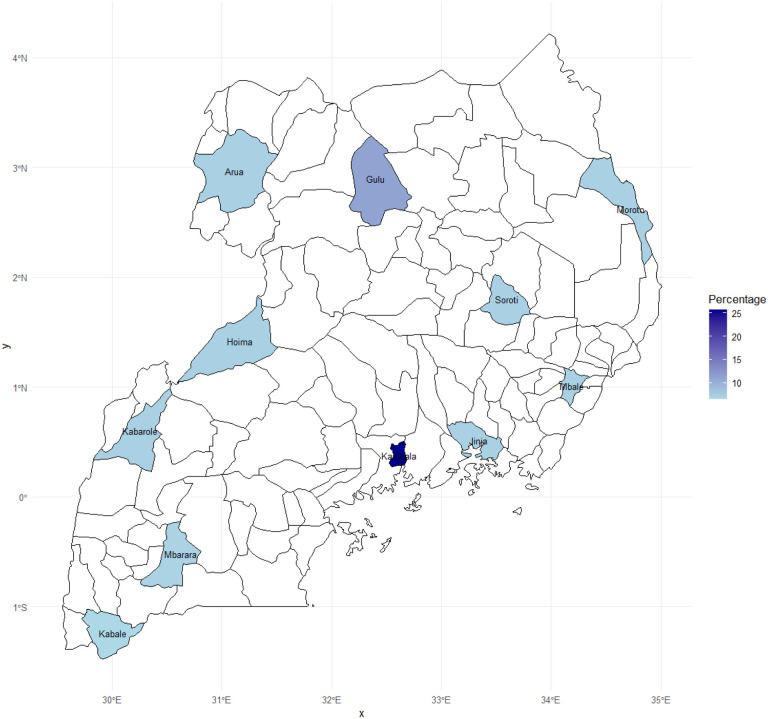
A map of Uganda showing the distribution of the study participants.

Seven hundred and eight (n = 708) responses were received (response rate = 94%). After removing duplicates and ineligible entries, data from 691 healthcare workers were analyzed (Table 1). The median age was 32 (interquartile range: 28–38). More than half were females (55.0%, n = 380) and nurses (57.6%, n = 398). Most participants worked in public health facilities (84.8%, n = 586) and resided in urban settings (92.0%, n = 636). Nearly half (45.2%) had attained at least an undergraduate degree, while one-third (32.6%, n = 225) provided emergency care services from the internal medicine department. Whereas 16.1% (n = 111) reported ever having contact with persons suspected or confirmed to have EVD in their lifetime, only three reported ever being diagnosed with the disease. The distribution of the study participants is shown in Fig. 1.

### Ebola Virus Disease Outbreak Awareness

**Figure 2:**
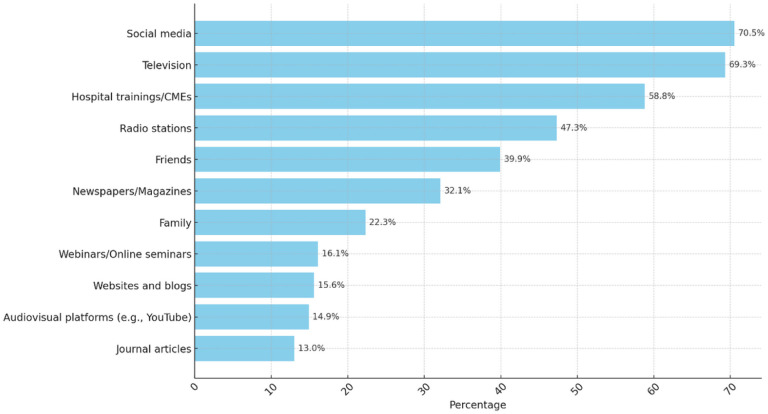
Healthcare workers’ source of information on the 2022 Ebola virus disease outbreak in Uganda

Most participants (93.3%, n = 645) were aware of the 2022 Ebola outbreak in Uganda. Social media (70.5%, n = 487), television (69.3%, n = 479), and hospital training (58.8%, n = 406) were the most frequent sources of information on EVD for more than half of the healthcare workers (Fig. 2). Only 34.4% (n = 238) had had training on EVD in the past year, with a median of 1 training (IQR: 1–2).

### Knowledge of Ebola Virus Disease

**Figure 3:**
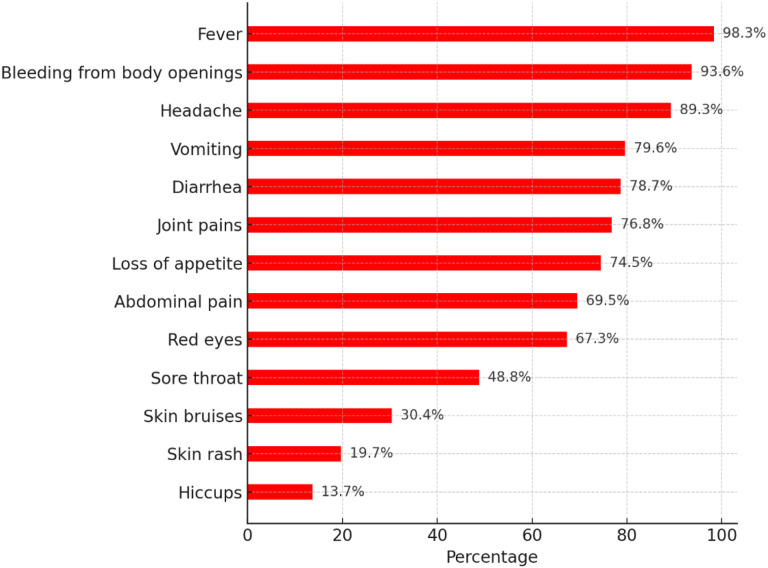
Knowledge of Ebola virus disease symptoms among healthcare workers in Uganda.

Most healthcare workers could identify the causative agents, transmission, and diagnosis of EVD (Table 2). However, more than 1 in 10 thought EVD was an airborne disease (16.1%) and could not be transmitted through contaminated objects, surfaces, and materials (12.7%). While most of the EVD symptoms were identified by the participants (Fig. 3), more than one-third (37.8%) thought that bleeding from orifices was the first symptom of EVD. More than half were not aware of effective treatments (52.0%) or vaccines for certain species of Ebola (50.5%). Up to 20.4% (n = 141) did not believe personal protective equipment could protect one from contracting EVD. The median knowledge score was 77.4% (IQR: 71.2% − 83.4%, range: 39.2% − 96.0%; scale internal reliability – 0.63). Based on Bloom’s cut-off, 39.1% (n = 270) had good knowledge (scores between 80–100%), 55.7% (n = 385) had moderate knowledge (60–79%), and only 5.2% (n = 36) had poor knowledge of EVD.

### Factors Associated with EVD Knowledge

In bivariate analysis (Table 3), healthcare workers from Eastern (β: −5.18, 95% CI: −7.18 to −3.19, p < 0.001) and Northern Uganda (β: −4.29,95% CI: −6.19 to − 2.39, p < 0.001) had significantly lower scores compared to those from Central Uganda. EVD knowledge scores were also significantly higher among males (β: 2.79,95% CI: 1.41 to 4.17, p < 0.001), doctors (β:6.44,95%CI:5.01 to 7.87, p < 0.001), and urban residents (β: 3.06,95% CI: 0.50 to 5.61, p = 0.019). Furthermore, EVD knowledge scores significantly increased with the level of education, with participants having a diploma (β: 4.05,95% CI: 2.16 to 5.94, p < 0.001), undergraduate (β: 8.83, 95% CI:6.89 to 10.77, p < 0.001), and postgraduate degrees (β: 11.08,95% CI: 8.68 to 13.48, p < 0.001) having higher scores compared to certificate holders. HCWs in internal medicine (β: 2.00, 95% CI: 0.52 to 3.47, p = 0.008) had significantly higher scores than their counterparts working in other departments, while those in obstetrics and gynecology had significantly lower scores (β: −3.75, 95% CI: −5.46 to − 2.03, p < 0.001). Furthermore, those who had had contact with a patient with suspected or confirmed EVD had twice as high scores (β: 2.00, 95%CI: 0.11 to 3.89, p = 0.038). Finally, having previous training on EVD (β: 2.27, 95%CI: 0.96 to 3.58, p = 0.001) and accessing EVD information from social media (β: 2.52, 95%CI: 1.16 to 3.87, p < 0.001) were significantly associated with higher knowledge scores.

At multilevel linear regression (Table 3), doctors (β: 2.55, 95% CI: 0.35 to 4.74, p = 0.023) and healthcare workers with longer professional experience in years (β: 0.21, 95% CI: 0.03 to 0.39, p = 0.024) significantly had higher EVD knowledge scores. Level of education also maintained a significant association with an increasing trend in EVD knowledge scores among those with diplomas (β: 3.37, 95% CI: 1.49 to 5.25, p < 0.001), undergraduate degrees (β: 6.45, 95% CI: 4.11 to 8.79, p < 0.001), and postgraduate degrees (β: 7.13, 95% CI: 4.01 to 10.25, p < 0.001), compared to certificate holders. In addition, HCWs who had previous EVD training (β: 2.27, 95% CI: 0.96 to 3.59, p = 0.001) and those who accessed EVD information from social media (β: 2.52, 95% CI: 1.17 to 3.88, p < 0.001) had more than twice higher EVD knowledge scores. However, healthcare workers in the obstetrics and gynecology department had significantly lower EVD knowledge scores (β: −1.90, 95% CI: −3.47 to − 0.32, p = 0.018).

### Attitudes Towards Ebola Virus Disease

**Figure 4:**
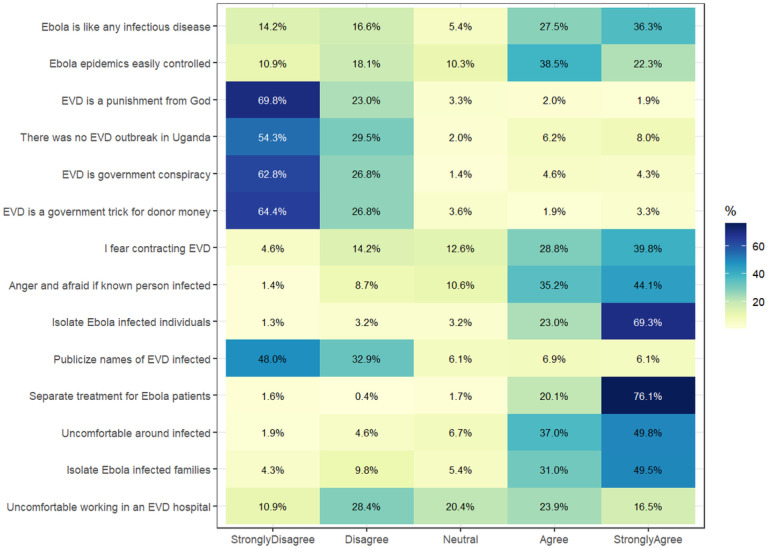
Perceptions of healthcare workers in Uganda towards Ebola virus disease.

Healthcare workers in this study generally held diverse perceptions towards the EVD outbreak in Uganda (Fig. 4). Up to 60.8% believed the EVD epidemics could be controlled easily, while 92.3% agreed that patients with EVD should be isolated. About 86.8% reported being uncomfortable near patients with suspected or confirmed EVD, while 40.4% reported being uncomfortable with working in a hospital with an Ebola treatment unit. Only 3.9%, 8.9%, and 14.2% believed that EVD was a punishment from God, a government conspiracy, and that there was no EVD outbreak in Uganda in 2022, respectively. Some 5.2% of healthcare workers believed that the EVD outbreak was a trick by the government to get funds from donors.

### Practices Related to EVD Outbreak

More than half of the healthcare workers reported consistently wearing personal protective equipment (PPEs) while caring for all patients (57.9%). In comparison, 66.1% reported doing so only when caring for patients with symptoms of EVD (Table 4). The majority (72.4%) reported disinfecting their workspaces daily, while 82.1% reported consistently observing the five moments of handwashing while caring for patients with EVD symptoms. Only 14% (n = 97) reported that they would never accept taking care of a patient with signs and symptoms suggestive of EVD. Nearly 46.0% (n = 318) reported never attending EVD-related trainings or workshops after the outbreaks. Up to 40% of the HCWs were uncomfortable taking care of patients suspected of having EVD.

### Training Needs

Nearly all the participants (99.6%) were interested in additional EVD training, with 44.6% very interested. Physical training mode was the most preferred (51.5%), followed by a hybrid format (38.2%). Only 10.3% preferred online training only. Healthcare workers were most interested in receiving training on EVD management (92.9%), infection prevention and control (87.1%), clinical presentation (84.1%), vaccination (82.6%), and diagnosis (80.5%; Table 5).

## Discussion

The Ebola virus disease remains a persistent public health concern, particularly in regions where it is endemic, such as parts of the Democratic Republic of Congo (DRC). Uganda, DRC’s neighboring country, bears a continuous threat of potential EVD outbreaks, having had more than five in the past two decades. This dilemma stresses the necessity for ongoing outbreak preparedness and the reinforcement of global health security, as highlighted by the 2022 Uganda Ebola outbreak. Our study sought to evaluate the readiness of emergency healthcare workers (HCWs) in 14 secondary and tertiary Ugandan hospitals and identify their specific training needs related to EVD. Results indicated that HCWs generally possess moderate to good EVD knowledge, with an average score of 77.4%. Despite a minority displaying poor attitudes, most demonstrated effective EVD infection prevention and control practices. Finally, HCWs in our study were strongly interested in additional EVD training.

The median knowledge score in our study closely aligns with that reported in Saudi Arabia [19] but surpasses the 52.0% reported in Iran [20]. Notably, 39.1% of HCWs in our study had good EVD knowledge, similar to findings in Nigeria [21] and Italy [22] but lower than in Pakistan [23], Sierra Leone [24], and Nigeria [25, 26]. Our participants had a good understanding of the etiology, transmission, diagnosis, management, and prevention of EVD, similar to studies in DRC [27], Nigeria [21, 28], and Romania [29]. However, HCWs in Guinea [30], Saudi Arabia [19], and South India [31] had poor knowledge of EVD management and transmission. A higher number of participants recognized the sexual transmission of EVD, markedly higher than 36% in Pretoria [32]. Conversely, misconceptions about EVD being airborne were less prevalent in our study than in Sudan [33]. Interestingly, while less than 1 in 10 of our study’s participants mistakenly believed EVD was bacterial, a notable 22.4% thought antibiotics could cure it, a belief similarly observed in Northwestern Ethiopia (28.4%) [16]. These variations in EVD knowledge among healthcare workers globally suggest a complex interplay of contextual factors. The fact that Uganda has experienced over five EVD epidemics in 20 years might have contributed to the HCW knowledge as a matter of vigilance.

Educational and training differences are essential, with varying curricula and emphasis on infectious diseases like EVD shaping knowledge levels. In our study, EVD knowledge increased significantly with HCWs’ level of education. Additionally, HCWs who had attended EVD training in the past year had knowledge scores that were more than twice as high. This finding is consistent with other studies in Romania [29], Saudi Arabia [19], and Guinea [24], where HCWs trained in EVD had higher knowledge. The focus of public health policies and allocating resources towards EVD education in different countries contribute to these knowledge gaps. During the EVD outbreak in Uganda, HCWs were trained by the Ministry of Health, Uganda, with support from multiple local and international non-governmental organizations, including the WHO, USAID, and UNICEF, among others [34]. Previous trainings had also been conducted for HCWs in the Ugandan districts bordering Eastern Congo, where the most significant threats were, with promising findings on knowledge and skills gained [14]. While knowledge of etiology and diagnosis was high in our study, there were pertinent gaps in infection prevention and control, with more than 20% believing that handwashing and PPEs do not protect one from getting infected with EVD. The gaps in infection prevention and control (IPC) for EBV may stem from the lack of exposure to practical drills, such as outbreak simulations, encompassing screening and outbreak management. We recommend continuous, pragmatic IPC training for HCWs to counteract misconceptions, with a preference for blended sessions incorporating physical modules, as indicated by over one-third of study participants.

Secondly, access to current, reliable medical information and resources also plays a crucial role; limited access can result in reliance on outdated or incorrect information. In our study, social media was the most frequent source of information on EVD among 70.5% of Ugandan HCWs and significantly had twice as much EVD knowledge. This can be attributed to the widespread accessibility of portable phones, which provide cost-effective access to news updates on outbreaks and academic content, including theory and practice videos, across more platforms than traditional T.V./Radio. A similar trend has also been reported in Ethiopia [16], Northeast Nigeria [35], and Romania [29], as opposed to the television and the medical institute ranking first, respectively. The role of social media in disseminating information during outbreaks is controversial and has had mixed effects [36]. While it has become an essential and indispensable communication medium, especially among young healthcare workers and the general population, it is also a significant source of misinformation, potentially causing an infodemic, especially when not regulated [36]. During the early days of the deadly EVD outbreak in Western Africa in 2014, 19% − 24% of social media posts were on health information, while 2% consisted of misinformation [37]. In the U.S., up to 10% of Ebola-related tweets contained false information [38]. Myths such as salty water and nano silver as potential treatments for EVD, reported among HCWs [39, 40], were spreading through social media [37]. However, social media can also be important in dispelling such myths and misinformation [36, 41]. While television and radio speeches were widely used in Uganda, they were not significantly associated with higher knowledge among HCWs in our study. We recommend that governmental and non-government agencies actively consider adopting social media communications to disseminate information regarding outbreaks to HCWs. Innovative ways such as Twitter hashtags, social media groups, Telegram channels, TikTok, or Instagram reels could be explored with input from communication specialists and further studied for effectiveness, even in the general population, with caution towards privacy and confidentiality [42].

Knowledge differences could be attributed to exposure to EVD outbreaks among the HCWs. Countries with firsthand experience of EVD outbreaks, such as the Democratic Republic of Congo [6], Uganda [43], and Sierra Leone [44], are likely to possess more practical understanding, enhancing their healthcare workers’ knowledge. This direct exposure to EVD cases often necessitates a more immediate and in-depth learning experience, likely improving their knowledge and preparedness. The exposure may have provided health workers with unique, hands-on experiences that are not replicated through theoretical learning alone. Such experiences can deeply instill knowledge about EVD symptoms, transmission, and management. In our study, 16.1% had contact with persons suspected or diagnosed with EVD, and it was associated with higher EVD knowledge scores at bivariate analysis but lost significance after adjusting for potential confounders. This suggests that while direct exposure to EVD cases is an influential factor, it operates within a complex network of other variables contributing to knowledge levels. Factors such as educational background, access to resources, training programs, and personal motivation to stay informed about infectious diseases might play equally significant roles in shaping a healthcare worker’s knowledge base.

Sociocultural beliefs, myths, and misinformation also significantly impact perceptions and understanding of EVD among HCWs, leading to varied beliefs about its transmission and treatment [45]. While these were less prevalent in our study, their impact cannot be underestimated and deserves further exploration, especially using qualitative methods. However, we did not assess this as it was not primarily the objective of this study. Additionally, the specific demographics of survey participants, such as their specialization, can influence the EVD knowledge among HCWs. In our study, doctors had higher EVD knowledge scores than other professional cadres, consistent with studies in Sudan [33] and Nigeria [26, 28]. Only one study in Northeast Ethiopia found that doctors had lower EVD knowledge than allied health professionals, although this was not significant [16]. This can be attributed to differences in the medical curriculum, which emphasizes virology during pre-medical training while allied health professionals focus on prevailing illnesses. Given Uganda’s low doctor-patient ratio of 1:25,725, medical doctors could be utilized to conduct training and support supervision for lower-level health facilities that clinical officers and nurses majorly serve.

In our study, HCWs with more extended professional experience also had significantly higher EVD knowledge scores, in line with studies in Pakistan [46] and South India [31]. However, this was not significant in two studies from Nigeria [21, 26], DRC [27], Iran [20], and Romania [29]. While these findings are inconsistent and inconclusive, there are several counterarguments to consider regarding the role of professional experience in influencing knowledge of EVD. Longer professional experience may be associated with cumulative learning, where healthcare workers (HCWs) acquire more comprehensive knowledge over time through continued education and practical experience. This can be particularly true in fast-evolving fields like infectious diseases, where staying updated with the latest information and guidelines is crucial. However, more experience does not necessarily equate to updated knowledge. In rapidly changing fields, information learned at the beginning of one’s career may become outdated. Newer HCWs might be more recently trained with current information and guidelines, giving them an edge in their EVD knowledge. Moreover, the motivation to continuously learn and stay updated can vary widely among individuals, regardless of their professional years.

Finally, the contrast in knowledge among HCWs in the obstetrics and gynecology department compared to those in the internal bivariate analysis did not hold after adjusting for confounders. This disparity could be attributed to several factors. Firstly, the focus of training and continuing education in different specialties varies; internal medicine often deals more directly with infectious diseases, including EVD, leading to more in-depth coverage in their training and practice. In contrast, obstetrics and gynecology may prioritize other areas of women’s health, with less emphasis on general infectious diseases like EVD. Additionally, the nature of exposure and the perceived risk of encountering EVD can influence the level of knowledge. Internal medicine departments might be more likely to encounter a variety of infectious diseases, thereby necessitating a broader knowledge base. Conversely, in obstetrics and gynecology, the perceived risk of encountering EVD may be lower, potentially leading to less emphasis on EVD education and awareness. However, it’s noteworthy that these differences were not significant after adjusting for confounders, suggesting that factors such as overall work experience, access to training, and individual motivation to stay informed could also play crucial roles in determining EVD knowledge levels across different specialties.

Our study is the first and the largest one to comprehensively assess clinical healthcare workers’ preparedness in Uganda to manage EVD and evaluate training needs. This study also recruited participants from Uganda’s 15 major secondary and tertiary health facilities. However, there are some limitations. First, there was only one private facility and two private-not-for-Profit facilities, limiting its generalization to only public health facilities in Uganda. Secondly, we used consecutive non-random sampling methods that might not adequately represent the entire population of healthcare workers in Uganda, as they tend to include participants who are more readily accessible or willing to participate. Thirdly, our hierarchical model showed that healthcare facilities strongly moderated EVD knowledge among the participants (β: 6.80, 95% CI: 2.81 to 16.52, p < 0.001). However, we could not perform additional facility-level analysis due to multiple hospitals with smaller sample sizes. Lastly, while the study adjusted for several confounders, other unmeasured variables could influence the results, such as access to ongoing professional development, personal motivation, or specific hospital policies and practices regarding infectious disease management.

## Conclusion

Our findings reveal a generally moderate to good level of knowledge about Ebola virus disease among healthcare workers in Uganda. This was associated with longer professional experience, a higher level of education, being a medical doctor, the hospital department, previous EVD training, and receiving information from social media. While attitudes were generally positive, significant practice gaps regarding infection prevention and control were identified. We recommend that health policymakers and hospital administrators prioritize targeted strategies for adaptive, high-impact training to bridge practice gaps in infection prevention and control to enhance the overall preparedness and response to EVD and similar infectious disease outbreaks. Future research should explore the impact of innovative educational interventions and the role of social media in information dissemination, particularly in specialties and regions where knowledge gaps exist.

## Figures and Tables

**Table 1:** Social and demographic characteristics of the study participants.

Variables (N = 691)	Frequency (%)
**Age in years: median (interquartile range)**	32.0 (28.0–38.0)
**Age Category**	
Youth	455 (65.8)
Adults	232 (33.6)
Elderly	4 (0.6)
**Professional Cadre Group**	
Clinical Officer	62 (9.0)
Doctor	231 (33.4)
Nurse/Midwife	398 (57.6)
**Sex**	
Female	380 (55.0)
Male	311 (45.0)
**Hospital Ownership**	
Public	586 (84.8)
Private-Not-For-Profit	82 (11.9)
Private	23 (3.3)
**Hospital Region**	
Central	177 (25.6)
East	147 (21.3)
North	176 (25.5)
West	191 (27.6)
**Nature of current residence**	
Rural	55 (8.0)
Urban	636 (92.0)
**Highest level of education**	
Certificate	113 (16.4)
Diploma	266 (38.5)
Undergraduate degree	225 (32.6)
Postgraduate	87 (12.6)
**Professional experience in years: median** (interquartile range)	6.0 (3.0–10.0)
**Department (multiple responses accepted)**	
Internal medicine	225 (32.6)
Pediatrics	215 (31.1)
Surgery	179 (25.9)
Obstetrics and Gynecology	138 (20.0)
Acute and Emergency	42 (6.1)
Outpatient Department	20 (2.9)
Intensive Care Unit or Anaesthesia	15 (2.2)
**Diagnosed with Ebola virus disease (EVD)**	
No	688 (99.6)
Yes	3 (0.4)
**Contact with a person diagnosed or suspected to have EVD.**	
No	580 (83.9)
Yes	111 (16.1)

**Table 2:** Knowledge of healthcare workers in Uganda on Ebola virus disease.

Statement	True: n (%)	False: n (%)
Ebola is a disease caused by microorganisms classified under the kingdom of Bacteria	65 (9.4)	626 (90.6)
EVD belongs to the group of organisms that are classified as viral hemorrhagic fevers	685 (99.1)	6 (0.9)
The following are EVD species that cause human disease.		
Zaire	522 (75.5)	169 (24.5)
Gulu	55 (8.0)	636 (92.0)
Rakai	16 (2.3)	675 (97.7)
Sudan	423 (61.2)	268 (38.8)
Bundibugyo	242 (35.0)	449 (65.0)
Mubende	153 (22.1)	538 (77.9)
Tai Forest	62 (9.0)	629 (91.0)
Zika	81 (11.7)	610 (88.3)
None of the above	76 (11.0)	615 (89.0)
Fruit bats are the natural reservoir host for Ebola	576 (83.4)	115 (16.6)
Body fluids can transmit EVD from an infected person	683 (98.8)	8 (1.2)
EVD cannot be transmitted by objects and materials contaminated with body fluids	88 (12.7)	603 (87.3)
EVD is an airborne disease spread through inhalation of air from infected persons	111 (16.1)	580 (83.9)
One cannot get EVD by touching the dead bodies of Ebola patients	90 (13.0)	601 (87.0)
EVD can be acquired directly from infected animals	660 (95.5)	31 (4.5)
Mosquito bites can transmit EVD from an infected person to an uninfected person	68 (9.8)	623 (90.2)
One can get EVD by having sex with an infected person	646 (93.5)	45 (6.5)
A person with EVD cannot transmit the disease if they don’t have symptoms	182 (26.3)	509 (73.7)
Symptoms of EVD can appear between 2 to 21 days after contact with an infected person	662 (95.8)	29 (4.2)
The first symptom of EVD is bleeding from the mouth, nose, or ears only	261 (37.8)	430 (62.2)
There is currently no test for diagnosing EVD; therefore, only clinical presentation can be used	158 (22.9)	533 (77.1)
EVD can be diagnosed using a polymerase chain reaction (PCR) test	621 (89.9)	70 (10.1)
There are effective treatments for the treatment of some species of EVD	332 (48.0)	359 (52.0)
Antibiotics can be used to cure patients with EVD	155 (22.4)	536 (77.6)
The primary strategy of care for patients with EVD is supportive care	653 (94.5)	38 (5.5)
One can avoid contracting EVD by always wearing medical or surgical masks	347 (50.2)	344 (49.8)
EVD can be prevented by avoiding contact with the blood and body fluids of infected individuals	678 (98.1)	13 (1.9)
Washing hands after touching surfaces contaminated with Ebola virus cannot prevent the spread of the disease	215 (31.1)	476 (68.9)
There is currently an effective vaccine that can protect one from getting EVD	342 (49.5)	349 (50.5)
Using personal protective equipment accurately and consistently cannot protect one from EVD	141 (20.4)	550 (79.6)

**Table 3:** Factors associated with Ebola virus disease knowledge among healthcare workers in Uganda.

Variable Name	Crude Coefficient (95% CI)	P-value	Adjusted Coefficient (95% Cl)	P-value
**Hospital Region**				
Central	Reference		Reference	
East	−5.18 (−7.18, −3.19)	< 0.001	−2.38 (−7.87, 3.11)	0.396
North	−4.29 (−6.19, −2.39)	< 0.001	−0.33 (−5.05, 4.39)	0.892
West	−1.67 (−3.53, 0.19)	0.079	−0.35 (−5.57, 4.87)	0.896
**Public Hospital**				
No	Reference		Reference	
Yes	−0.98 (−2.91, 0.96)	0.322	−0.41 (−5.13, 4.32)	0.866
**Age**	0.05 (−0.03, 0.13)	0.212	−0.08 (−0.22, 0.06)	0.266
**Sex**				
Female	Reference		Reference	
Male	2.79 (1.41, 4.17)	**<** 0.001	1.18 (−0.24, 2.61)	0.104
**Profession Category**				
Nurse/Midwife	Reference		Reference	
Clinical Officer	0.84 (−1.53, 3.20)	0.487	−0.49 (−2.90, 1.92)	0.689
Doctor	6.44 (5.01, 7.87)	< 0.001	2.55 (0.35, 4.74)	0.023
**Location**				
Rural	Reference		Reference	
Urban	3.06 (0.50, 5.61)	0.019	1.64 (−0.59, 3.86)	0.15
**Education**				
Certificate (Ref.)	Reference		Reference	
Diploma	4.05 (2.16, 5.94)	**<** 0.001	3.37 (1.49, 5.25)	**<** 0.001
Undergraduate Degree	8.83 (6.89, 10.77)	**<** 0.001	6.45 (4.11, 8.79)	**<** 0.001
**Hospital Region**				
Postgraduate	11.08 (8.68, 13.48)	< 0.001	7.13 (4.01, 10.25)	< 0.001
**Professional experience (years)**	0.02 (−0.08, 0.12)	0.649	0.21 (0.03, 0.39)	0.024
**Internal Medicine**				
No	Reference		Reference	
Yes	2.00 (0.52, 3.47)	0.008	1.23 (−0.13, 2.59)	0.077
**Surgery**				
No	Reference		Reference	
Yes	−0.02 (−1.61, 1.57)	0.981	−1.01 (−2.40, 0.38)	0.156
**Obstetrics**				
No	Reference		Reference	
Yes	−3.75 (−5.46, −2.03)	< 0.001	−1.90 (−3.47, −0.32)	0.018
**Pediatrics**				
No	Reference		Reference	
Yes	−0.46 (−1.96, 1.04)	0.544	−0.26 (−1.61, 1.08)	0.704
**Emergency or acute care unit**				
No	Reference		Reference	
Yes	−0.71 (−3.62, 2.20)		−0.16 (−2.80, 2.49)	0.908
**Contact with a patient with Ebola.**				
No	Reference		Reference	
Yes	2.00 (0.11, 3.89)	0.038	−0.28 (−1.98, 1.42)	0.747
**Previous EVD training**				
No	Reference		Reference	
Yes	3.63 (2.19, 5.06)	< 0.001	2.27 (0.96, 3.59)	0.001
**Hospital Region**				
**Information from social media**				
No	Reference		Reference	
Yes	2.31 (0.80, 3.83)	0.003	2.52 (1.17, 3.88)	**<** 0.001
**Information from television**				
No	Reference		Reference	
Yes	0.70 (−0.81, 2.20)	0.364	0.88 (−0.45, 2.21)	0.193

**Table 4:** Ebola virus disease-related practices among healthcare workers in Uganda.

Practices	Frequency (%)
N	691
I wear personal protective equipment when taking care of all patients.	
Always	400 (57.9)
Sometimes	284 (41.1)
Never	7 (1.0)
I wear personal protective equipment only when caring for patients with symptoms of EVD.	
Always	457 (66.1)
Sometimes	102 (14.8)
Never	132 (19.1)
I do accept to take care of a patient once I notice signs and symptoms of EVD.	
Always	347 (50.2)
Sometimes	247 (35.7)
Never	97 (14.0)
I observe the five moments of handwashing while caring for patients with EVD symptoms.	
Always	567 (82.1)
Sometimes	108 (15.6)
Never	16 (2.3)
How often have you attended Ebola-related training or workshops after the outbreak?	
Daily	26 (3.8)
Monthly	41 (5.9)
Never	318 (46.0)
Rarely	284 (41.1)
Weekly	22 (3.2)
How often do you disinfect your workspace in light of the Ebola outbreak?	
Daily	500 (72.4)
Monthly	19 (2.7)
Never	31 (4.5)
Rarely	94 (13.6)
Weekly	47 (6.8)
How comfortable are you with treating patients suspected of having Ebola?	
Extremely comfortable	85 (12.3)
Somewhat comfortable	187 (27.1)
Neutral	142 (20.5)
Somewhat uncomfortable	153 (22.1)
Extremely uncomfortable	124 (17.9)

**Table 5:** Ebola virus disease training needs and preferences among healthcare workers in Uganda.

Training Preferences	Frequency (%)
N	691
How interested are you in receiving more training on EVD?	
Extremely interested	308 (44.6)
Very interested	290 (42.0)
Somewhat interested	51 (7.4)
Slightly interested	39 (5.6)
Not at all interested	3 (0.4)
What would be your preferred mode of training?	
Physical	356 (51.5)
Online	71 (10.3)
Hybrid	264 (38.2)
Which aspects of Ebola virus disease would you like to be trained on? (Multiple responses allowed)	
Management - treatment and providing care	642 (92.9)
Infection prevention and control	602 (87.1)
Clinical presentation - signs and symptoms	581 (84.1)
Ebola vaccination	571 (82.6)
Diagnosis	556 (80.5)
Transmission - how Ebola spreads	547 (79.2)
Epidemiology - distribution of Ebola	517 (74.8)
Body handling and burial practices after death	502 (72.6)
Biology of Ebola - basic science	474 (68.6)

## Data Availability

All data associated with this study has been adequately described in the manuscript. The original dataset and the STATA analysis do-file shall be freely available upon request from the corresponding author (Dr Ronald Olum, olum.ronald@gmail.com).

## Abbreviations

EVD: Ebola Virus Disease
HCWs: Healthcare Workers
RRH: Regional Referral Hospital
NRH: National Referral Hospital
PNFP: Private-not-for-Profit
IQR: Interquartile Range
CI: Confidence Interval
SARS-COV-1: Severe Acute Respiratory Syndrome Coronavirus 1
MERS-CoV: Middle East Respiratory Syndrome Coronavirus
CDC: Centers for Disease Control and Prevention
PCR: Polymerase Chain Reaction
PPEs: Personal Protective Equipment
